# Early Progressive Changes in White Matter Integrity Are Associated with Stroke Recovery

**DOI:** 10.1007/s12975-020-00797-x

**Published:** 2020-03-04

**Authors:** Daniela Pinter, Thomas Gattringer, Simon Fandler-Höfler, Markus Kneihsl, Sebastian Eppinger, Hannes Deutschmann, Alexander Pichler, Birgit Poltrum, Gernot Reishofer, Stefan Ropele, Reinhold Schmidt, Christian Enzinger

**Affiliations:** 1 Department of Neurology, Research Unit for Neuronal Plasticity and Repair, Medical University of Graz, Graz, Austria; 2 Department of Neurology, Medical University of Graz, Graz, Austria; 3 Department of Radiology, Division of Neuroradiology, Vascular and Interventional Radiology, Medical University of Graz, Graz, Austria

**Keywords:** Stroke, DTI, Corpus callosum, Prediction, Recovery, Longitudinal

## Abstract

Information on microstructural white matter integrity has been shown to explain post-stroke recovery beyond clinical measures and focal brain damage. Especially, knowledge about early white matter changes might improve prediction of outcome. We investigated 42 acute reperfused ischemic stroke patients (mean age 66.5 years, 40% female, median admission NIHSS 9.5) with a symptomatic MRI-confirmed unilateral middle cerebral artery territory infarction 24–72 h post-stroke and after 3 months. All patients underwent neurological examination and brain MRI. Fifteen older healthy controls (mean age 57.3 years) were also scanned twice. We assessed fractional anisotropy (FA), mean diffusivity (MD), axial (AD), and radial diffusivity (RD). Patients showed significantly decreased white matter integrity in the hemisphere affected by the acute infarction 24–72 h post-stroke, which further decreased over 3 months compared with controls. Less decrease in FA of remote white matter tracts was associated with better stroke recovery even after correcting for infarct location and extent. A regression model including baseline information showed that the modified Rankin Scale and mean FA of the genu of the corpus callosum explained 53.5% of the variance of stroke recovery, without contribution of infarct volume. Furthermore, early dynamic FA changes of the corpus callosum within the first 3 months post-stroke independently predicted stroke recovery. Information from advanced MRI measures on white matter integrity at the acute stage, as well as early dynamic white matter degeneration beyond infarct location and extent, improve our understanding of post-stroke reorganization in the affected hemisphere and contribute to an improved prediction of recovery.

**Electronic supplementary material:**

The online version of this article (10.1007/s12975-020-00797-x) contains supplementary material, which is available to authorized users.

## Introduction

Stroke represents a leading cause of a long-term disability in adults [1]. A better understanding of cerebral mechanisms associated with recovery holds promise to improve prediction of stroke recovery and outcome [2]. Especially, information on white matter integrity, derived from advanced MRI, such as diffusion tensor imaging (DTI), has been shown to explain post-stroke recovery beyond clinical measures [2–4]. DTI takes advantage of the diffusion of water in brain tissue within three main directions, which is decreased perpendicularly to the myelin sheaths and cell membranes of white matter axons [5, 6]. The most common metrics of DTI used are fractional anisotropy (FA) and mean diffusivity (MD) [5].

Information on disintegrity of white matter tracts in acute stroke patients, for instance of the corticospinal tract (CST) 12 h after stroke, has been shown to correlate well with motor function 90 days post-stroke and was superior to infarct volume and baseline clinical scores in outcome prediction [7]. In line with this, normal-appearing white matter integrity measured 24 to 72 h after symptom onset independently predicted functional outcome 1 year after stroke, in addition to demographic confounders, volumes of white matter hyperintensities, gray matter, and ischemic lesions [8].

More specifically, in particular, longitudinal DTI studies highlighted an association—in addition to focal damage from ischemic lesions—of close and remote secondary white matter degeneration with worse outcome [9–14]. These studies show that especially dynamic changes within the first weeks post-stroke are related to functional outcome. However, to date, only few longitudinal DTI studies investigated acute patients. These reported progressive decreases in white matter integrity within the first 3 months post-stroke which were associated with neurological recovery [11, 13]. A recent study investigating acute stroke patients with neglect highlighted that white matter remodeling directly induced by the stroke lesion should be distinguished from such processes evoked by remote network dysfunction [10].

A recent review reported that structural network connectivity, as assessed by DTI, might be more important for stroke outcome and recovery than the extent of the primary structural lesion, as it allows illustrating remote widespread microstructural white matter damage beyond focal damage [4]. Therefore, we aimed to assess (a) early perilesional and remote microstructural changes post-stroke and (b) the associations between lesion-independent white matter integrity changes and stroke recovery in a homogeneous sample of acute stroke patients with middle cerebral artery infarction.

## Patients and Methods

Data that support the findings of this study are available from the corresponding author upon reasonable request.

### Participants

We included 42 patients with a symptomatic MRI-confirmed ischemic infarction in the territory of the middle cerebral artery who had received a reperfusion therapy (intravenous thrombolysis and/or mechanical thrombectomy) according to local treatment guidelines and were 18–85 years of age. Exclusion criteria were a National Institute of Health Stroke Scale (NIHSS) score prior to reperfusion therapy > 20, contraindications for MRI, and severe premorbid cognitive impairment precluding participation in our study. All patients underwent a thorough neurological examination and comprehensive brain MRI at 3T post-treatment (24–72 h after symptom onset, baseline, BL) and at 3 months follow-up (FU) (Table 1). Baseline NIHSS (median = 3.5, IQR = 4, range = 0–11 vs. median = 2, IQR = 4, range = 0–9; *p* = 0.248) and modified Rankin Scale (mRS) scores (median = 4, IQR = 2, range = 1–5 vs. median = 4, IQR = 2, range = 0–5; *p* = 0.303) did not differ between patients with left and right hemispheric infarction. NIHSS (*p* = 0.182) and mRS at BL (*p* = 0.256) did not differ with respect to reperfusion therapy.

**Table 1:** Demographics, clinical, and MRI characteristics of the total cohort (*N* = 42), and comparisons regarding these variables in the subcohort of patients with available follow-up MRI at 3 months (*N* = 30)

	Total BL cohort (*N* = 42)	FU cohort (*N* = 30)
Age at baseline (years, SD)	66.5 (10.9)	66.3 (11.1)
Sex, *n* (% male)	25 (60%)	20 (67%)
Clinical characteristics, *n* (%)		
NIHSS (median, IQR, range) at admission	9.5 (11.0), 2–20	10.0 (11.0)
NIHSS (median, IQR, range) at discharge	1.0 (3.0), 0–9	1.5 (3.0)
NIHSS (median, IQR, range) at FU	–	1.0 (2.0), 0–4
i.v. thrombolysis only	11 (26%)	9 (30%)
Mechanical thrombectomy*	31 (74%)	21 (70%)
TICI 2b	11 of 31 (35%)	9 of 21 (43%)
TICI 3	20 of 31 (65%)	12 of 21 (57%)
Vascular risk factors		
Arterial hypertension	31 (74%)	22 (73%)
Atrial fibrillation	13 (31%)	11 (37%)
Hyperlipidemia	25 (59%)	17 (57%)
Diabetes mellitus	5 (12%)	4 (13%)
Smoking	14 (33%)	9 (30%)
MRI		
Symptom onset to baseline MRI (days, IQR, range)	1.0 (1.3), 1–4	1.0 (1.25), 1–4
FLAIR lesion size, cm^3^ (IQR, range)	7.57 (19.92), 0–114	8.46 (27.88), 0–114
WMH grade (IQR, range)	1.0 (1.0), 0–3	1.0 (1.0), 0–3
Lesioned hemisphere, *n* (% right)	18 (43%)	14 (47%)

*BL* baseline, *FU* 3-month follow-up, *NIHSS* National Institutes of Health Stroke Scale, *IQR* interquartile range

^*^13 patients additionally received i.v. thrombolysis

To control for stability and reproducibility of the imaging data, we assessed 15 healthy controls twice (mean age 57.3 years (± 10.9), 47% male).

### Brain MRI Acquisition

At BL and FU, all participants underwent brain MRI on the same 3 Tesla scanner (Prisma, Siemens Healthineers, Erlangen, Germany). The protocol included diffusion-weighted imaging (DWI, 1 × 1 × 5.5 mm, TR = 5000 ms; TE = 114 ms; *b* value = 1000), a high-resolution structural 3D scan by means of a T1-weighted MPRAGE sequence with 1-mm isotropic resolution (TR = 1900 ms; TE = 2.7 ms), T2-weighted (1 × 1 × 5 mm; TR = 690 ms; TE = 19.9 ms), FLAIR (1 × 1 × 3 mm; TR = 10,000 ms; TE = 95 ms), and diffusion (2 × 2 × 2 mm; TR = 2550 ms; TE = 89 ms; phase encoding direction anterior-posterior and posterior-anterior) sequences, as well as an intracranial 3D TOF angiography.

All MRI scans were reviewed by a neuroradiological expert (CE), assessing infarct location and extent as well as vessel recanalization status according to the thrombolysis in cerebral infarction (TICI) scale. Severity of white matter hyperintensities was graded using the Fazekas scale (0–3) [15]. No major intracranial bleeding post-reperfusion was observed in the study sample; only minimal signs of hemorrhagic transformation were present in 13 patients. Old lacunar infarcts (< 2 cm maximum diameter) were observed in five patients.

### Image Processing

**Fig. 1:**
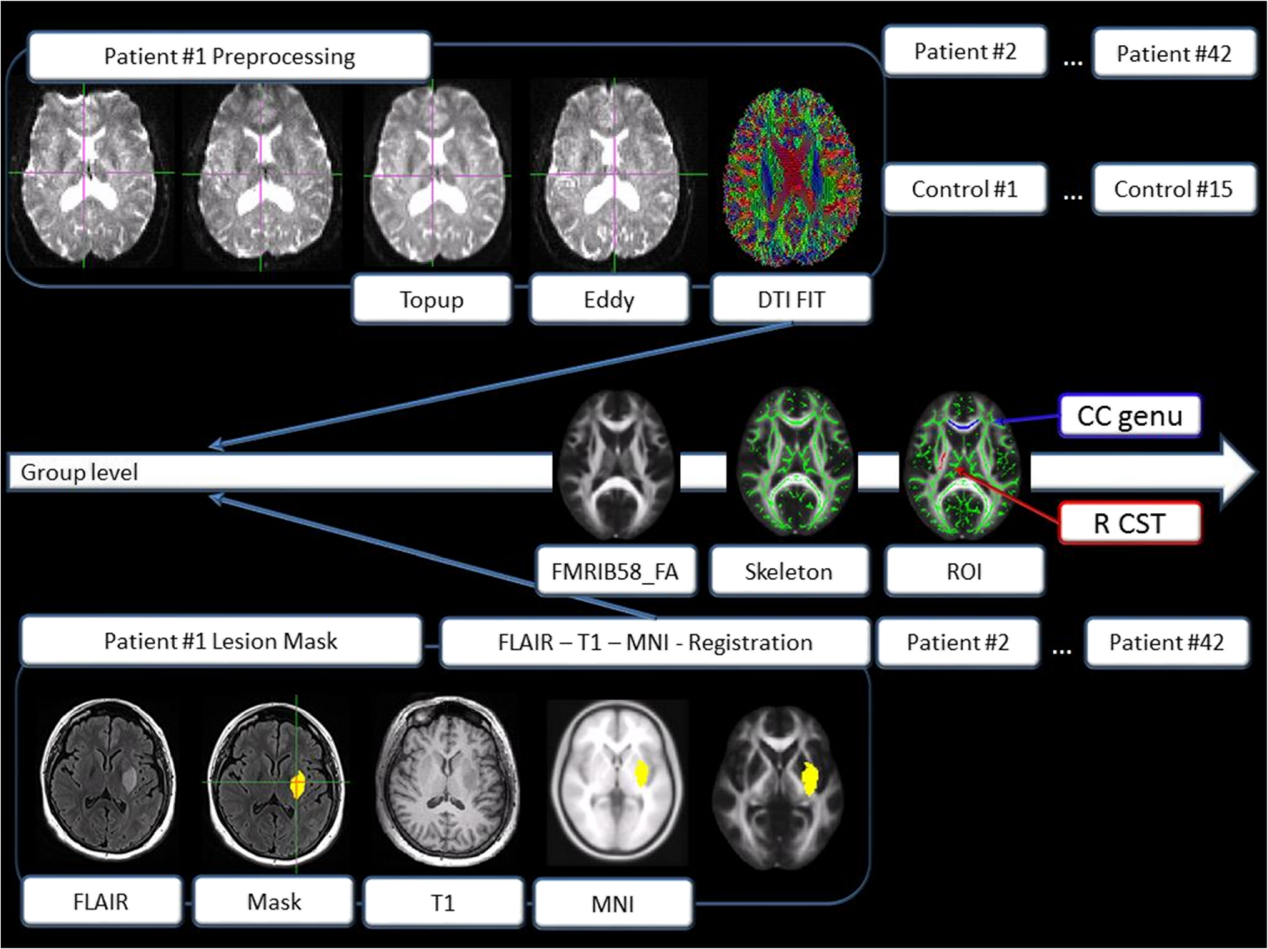
Processing flowchart, showing preprocessing steps and lesion registration for each individual. Group analyses were performed using the fMRIB58 FA template

Infarcts were defined as acute and related to the clinical finding using DWI (lesions with ADC restriction) by a blinded expert (CE). Infarcts were subsequently manually segmented on FLAIR images by DP, using information on lesion location from DWI images, and registered on the high-resolution T1-weighted MPRAGE scans to assess the location and extent of focal tissue destruction. For group comparisons, all images and infarcts were further registered to a standard template (MNI, see Fig. 1). Images from 18 patients with right hemispheric stroke were mirrored around the midline so that the lesioned hemisphere could be overlaid with patients with left hemispheric stroke, rendering the left hemisphere virtually lesioned in all patients [9, 11, 12, 16].

Diffusion tensor imaging (DTI) data was collected with reversed phase-encode blips, resulting in pairs of images with distortions going in opposite directions. From these pairs, the susceptibility-induced off-resonance field was estimated to correct for geometrical distortions using FSL (topup). A brain mask was created using BET (brain extraction tool). Eddy current-induced distortions were corrected with “eddy.” Maps for fractional anisotropy (FA), mean diffusivity (MD), and axial diffusivity (AD, along the axis of the fiber, 1st eigenvalue) were generated using the FDT (fMRIB’s Diffusion Toolbox, Version v 3.0). Radial diffusivity (RD) was generated using “fslmaths,” using the mean of the 2nd and 3rd eigenvalue [(L2 + L3)/2]. Subsequently, voxel-wise statistical analysis of FA data was carried out using TBSS (tract-based spatial statistics). Nonlinear registration into standard space was performed, using the FMRIB58_FA standard template. The same template was used to create the mean FA image. The FA skeleton was thresholded at 0.20 to include major white matter pathways but avoid peripheral tracts (vulnerable to inter-subject variability). Each subject’s FA map was then projected onto the mean skeleton. Voxel-wise cross-subject statistics (*p* > 0.05) by threshold-free cluster enhancement (TFCE), avoiding use of an arbitrary threshold for the initial cluster formation, was applied. TFCE allows to enhance cluster-like structures in an image without having to define an initial cluster-forming threshold or carry out a large amount of data smoothing [17]. We used non-parametric testing as implemented in “randomise” (5000 permutations), for calculating group and time contrasts. “Randomise” is a permutation method, which is used for inference (thresholding) on statistic maps when the null distribution is not known. Mean diffusivity, axial, and radial diffusivity were analyzed using TBSS and “randomise” in an analogous fashion. The anatomical location of significant clusters was determined by reference to the fiber tract-based atlas of human white matter (JHU ICBM-DTI-81 White-Matter Labels, JHU White-Matter Tractography Atlas, Juelich Histological Atlas), implemented in FSL. To assess significant changes of white matter integrity across 3 months beyond infarct location and extent, we included individual standardized and binarized infarct maps in the GLM (“randomise” options --vxl, --vxf).

As regional information on white matter integrity of the corpus callosum (CC) and corticospinal tract (CST) has been suggested to be especially informative for the prediction of stroke recovery [3, 18], we used the JHU White-Matter Labels atlas implemented in FSL, to define five regions of interest (ROIs): the genu, body and splenium of the corpus callosum (CC), and the right and left corticospinal tract (CST). An overview of the processing steps is shown in Fig. 1.

### Statistical Analysis

Demographic, clinical, and MRI data were analyzed with the Statistical Package of Social Science (IBM SPSS Statistics 23). The level of significance was set at 0.05. The Kolmogorov-Smirnov test assessed normality of data distribution. Groups were compared by the chi-square test (for nominal data), the Mann–Whitney *U* or Wilcoxon test (for non-normally distributed variables), or *t* test (for continuous, normally distributed variables). Correlation analysis was performed using the Spearman or Pearson correlation.

A hierarchical linear regression analysis was performed to identify markers associated with recovery (change in NIHSS score), including WMH score at baseline, degree of disability at baseline (mRS), infarct volume, and DTI data.

## Results

### Post-Treatment Scan: White Matter Integrity in Acute Stroke Patients Compared with Controls

**Fig. 2:**
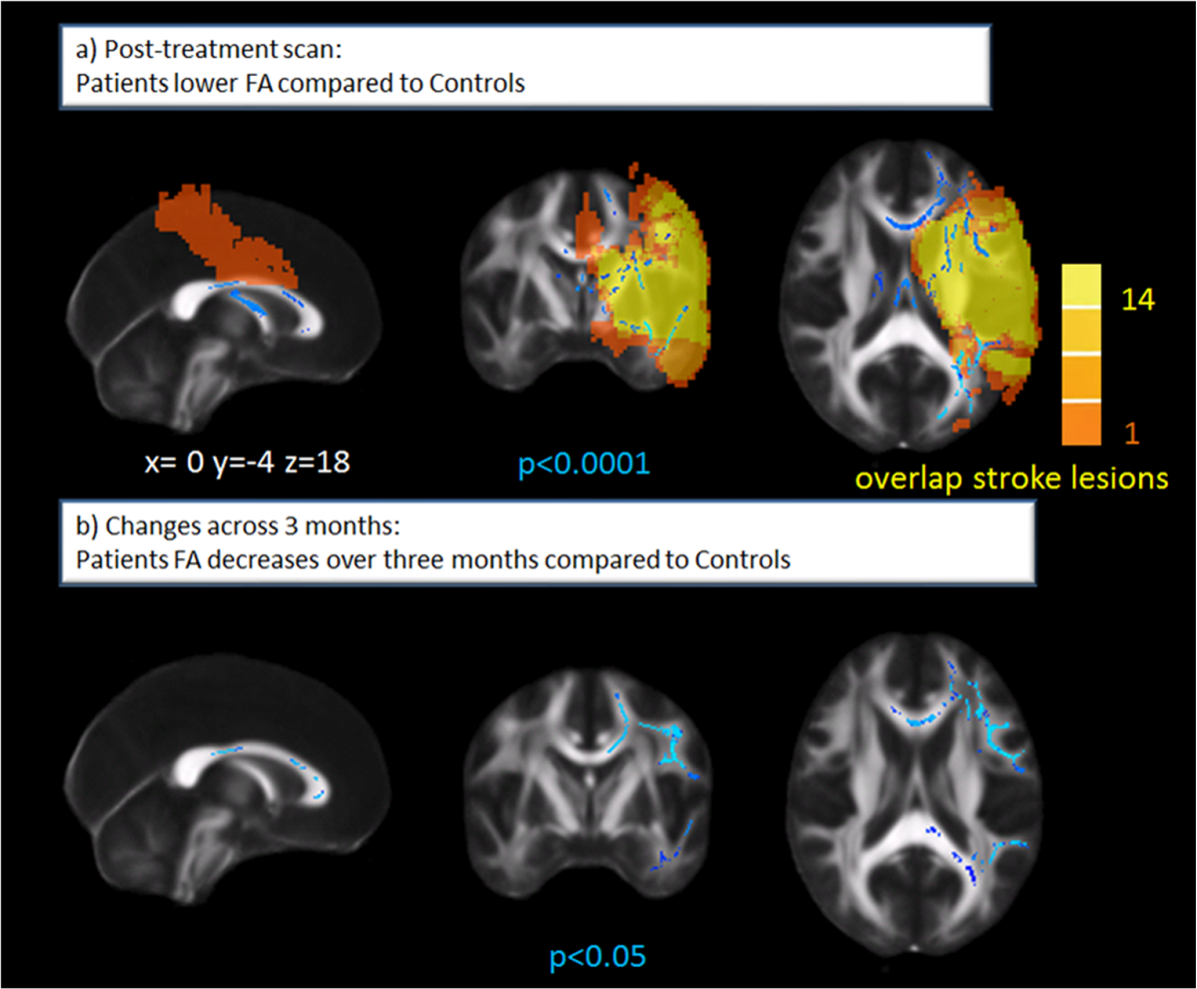
**a** Lower white matter integrity in stroke patients compared with controls at post-treatment scan (24–72 h after symptom onset) shown in blue. Note that even in this homogeneous sample, there is substantial variability in the extent of the ischemic lesions ranging from 1 to 14 overlapping lesions presented in orange-yellow. FA, fractional anisotropy. **b** Significant decreases in white matter integrity (assessed by FA) in patients compared with controls over 3 months post-stroke

Voxel-wise whole brain comparison of white matter integrity showed significantly lower FA values in the lesioned (left) hemisphere in stroke patients compared with healthy controls. Values were lower in the corpus callosum (CC), the left superior and inferior longitudinal fasciculi, left corticospinal tract (CST), left anterior thalamic radiation, left inferior fronto-occipital fasciculus, left uncinate fasciculus, left forceps major, and minor in patients compared with controls (Fig. 2a).

Accordingly, significantly higher RD values were observed in patients compared with controls in the abovementioned regions, with additionally higher values in the right inferior fronto-occipital fasciculus, right major and minor forceps, right anterior thalamic radiation, and right uncinate fasciculus. A trend for higher MD values (0.10) was observed in the CC and posterior corona radiata. No significant differences in AD were observed at the post-treatment scan.

Extracted mean values of white matter integrity (mean FA, MD, AD, and RD values) for the five ROIs (CC genu, body and splenium, left and right CST) did not significantly differ between acute patients compared with controls at post-treatment scan (Online Resource Table S1).

### Longitudinal Changes of White Matter Integrity in Patients Compared with Controls

Out of 42 patients that participated in our study, MRI data of 30 patients in sufficient quality was available for both BL and FU analyses after MRI data quality checks (e.g., motion artifacts and registration). Six patients were excluded due to recurrent stroke (*N* = 2) or severe medical worsening before the follow-up (FU) visit (*N* = 4). Duration to follow-up MRI did not significantly differ between patients (median 103 days, IQR 33, range = 55–176) and controls (*N* = 15, median 91 days, IQR 12, range = 56–147). Delta NIHSS (stroke recovery) did not differ between left and right hemispheric strokes (*p* = 0.334).

Across 3 months, we observed no differences in white matter integrity for healthy controls, attesting to the stability and reliability of the MRI data and analyses.

Whole brain comparison of white matter integrity difference maps showed significant FA decreases in patients compared with controls within the first 3 months post-stroke (Fig. 2b). These concerned the CC, left superior longitudinal fasciculus, left inferior fronto-occipital fasciculus, and the left forceps major and minor. Accordingly, significant MD increases in patients compared with controls within the first 3 months post-stroke were observed in the bilateral CC, bilateral CSTs, bilateral superior longitudinal fasciculi, forceps minor, and forceps major. Axial diffusivity significantly increased in the left superior corona radiata and left inferior fronto-occipital fasciculus and decreased in the left cerebral peduncle, posterior limb of internal capsule, and right forceps major. Widespread bilateral RD increases were observed within 3 months for patients (CC, CST, superior frontal longitudinal fasciculus, inferior fronto-occipital fasciculus, forceps minor and major).

Extracted mean scores of changes in white matter integrity showed that MD increased significantly in the CC splenium and left CST, and RD increased significantly across all ROIs in patients compared with controls across 3 months (Table 2).

**Table 2:** Comparison of extracted mean changes in white matter integrity between patients and controls from baseline (in patients: post-treatment) scan to the 3-month follow-up. *CC* corpus callosum, *LCST* left corticospinal tract, *RCST* right corticospinal tract. Mann–Whitney *U* Test. Bonferroni-adjusted level of significance = 0.01

	Patients (*N* = 30)	Controls (*N* = 15)	*p* value
diffFA			
CC genu	− 0.01001 (0.03122)	0.00330 (0.01575)	0.060
CC body	− 0.01163 (0.04321)	0.00245 (0.02741)	0.071
CC splenium	− 0.00444 (0.02276)	0.00306 (0.01624)	0.041
LCST	− 0.01131 (0.02731)	− 0.00260 (0.01617)	0.048
RCST	− 0.00693 (0.02091)	− 0.00420 (0.01183)	0.118
diffMD			
CC genu	0.00005 (0.00019)	− 0.00001 (0.00004)	0.028
CC body	0.00005 (0.00019)	− 0.00000 (0.00003)	0.011
CC splenium	0.00004 (0.00021)	− 0.00000 (0.00002)	*0.004*
LCST	0.00004 (0.00008)	− 0.00000 (0.00002)	*0.001*
RCST	0.00002 (0.00019)	− 0.00001 (0.00003)	*0.006*
diffAD (mm^2^ s^−1^)			
CC genu	0.000002 (0.000097)	− 0.000001 (0.000047)	0.754
CC body	0.000018 (0.000086)	0.000005 (0.000044)	0.248
CC splenium	0.000016 (0.000073)	0.000001 (0.000025)	0.092
LCST	− 0.000010 (0.000090)	− 0.000008 (0.000026)	0.791
RCST	0.000007 (0.000067)	0.000001 (0.000041)	0.149
diffRD (mm^2^ s^−1^)			
CC genu	0.00004 (0.00017)	− 0.00000 (0.00004)	*0.007*
CC body	0.00007 (0.00029)	− 0.00001 (0.00004)	*0.007*
CC splenium	0.00005 (0.00029)	− 0.00000 (0.00002)	*0.004*
LCST	0.00006 (0.00011)	− 0.00001 (0.00003)	*0.001*
RCST	0.00002 (0.00024)	− 0.00000 (0.00002)	*0.003*

All significant *p*-values are now highlighted in italics

Significantly, decreased mean FA in the CC body and increased MD in the CC and left CST as well as increased RD in all ROIs were observed in patients compared with controls at the 3 months follow-up (Online Resource Table S1).

### Post-Treatment or Follow-up Scan DTI Parameters and Stroke Recovery

Cross-sectional analyses showed trends for associations (*p* < 0.05) with higher mean FA values, as well as lower MD or RD values, of the genu of the CC at the post-treatment scan, correlating with better stroke recovery (delta NIHSS; Online Resource Table S2). None of these correlations remained significant after applying the Bonferroni-corrected level of significance 0.01. There were no other significant correlations at either time points.

FA did not differ with respect to reperfusion therapy (left CST, *p* = 0.084; right CST, *p* = 0.673; genu of the CC, *p* = 0.540; body of the CC, *p* = 0.736; splenium of the CC, *p* = 0.993).

There also were no significant associations between the whole brain white matter integrity on the post-treatment scan or follow-up scan and stroke recovery.

### Short-term White Matter Integrity Changes and Stroke Recovery

**Fig. 3:**
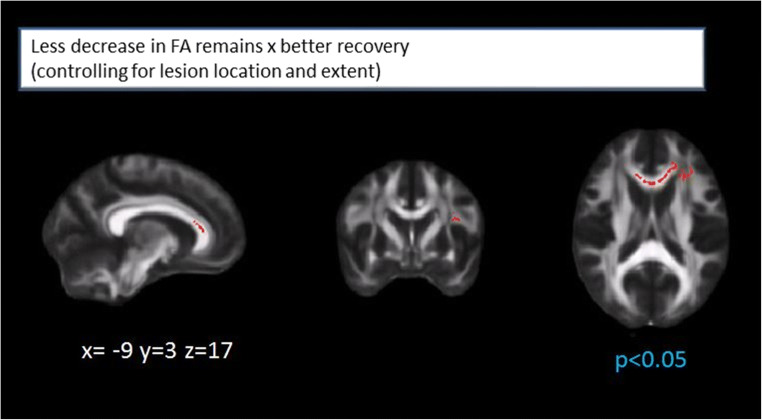
Less decrease in white matter integrity (assessed by FA values) in the genu of the corpus callosum and the left inferior fronto-occipital fasciculus (anterior part). Both remain associated with better stroke recovery (delta NIHSS) after correction for lesion location and extent

Less decrease in left hemispheric FA, specifically in the superior longitudinal fasciculus, the genu and body of the CC, the inferior longitudinal fasciculus and inferior fronto-occipital fasciculus, was associated with better recovery. Less decrease in white matter integrity (assessed by FA) in the genu of the CC and the left inferior fronto-occipital fasciculus (anterior part) remained associated with stroke recovery, even after correcting for infarct location and extent (Fig. 3).

Short-term changes of MD, AD, or RD were not significantly associated with stroke recovery.

We therefore chose to include information on FA of the genu of the CC (baseline and changes) in our prediction model.

### Prognostic Value of White Matter Integrity

A regression model including WMH score, mRS, infarct volume, and mean FA of the genu of CC at baseline explained 53.5% of variance in stroke recovery (delta NIHSS). There was a positive effect of mRS (βj = 0.476, *p* = 0.001) explaining 30.3% of variance and a positive effect of FA of genu of the CC (βj = 0.374, *p* = 0.004, higher baseline FA predicted better recovery) explaining an incremental percentage of 23.2 of variance. Infarct volume and WMH score did not significantly contribute to the prediction.

A regression model including WMH score, mRS, and infarct volume at baseline, as well as changes in white matter integrity (FA) in the genu of the corpus callosum explained 50.1% of variance in stroke recovery. There was a positive effect of mRS (βj = 0.498, *p* < 0.05), explaining 31.6% of variance, an incremental effect of infarct volume (βj = 0.498, *p* < 0.05, incremental *R*^2^ = 10.9%), and white matter changes (βj = 0.325, *p* < 0.05, incremental *R*^2^ = 7.6%, more decrease in FA predicting worse recovery).

## Discussion

This longitudinal DTI study shows that both acute and early dynamic white matter changes within 3 months post-stroke are associated with stroke recovery after middle cerebral artery occlusion, independent from infarct location and extent. Although impaired white matter integrity was already present 24–72 h after symptom onset compared with healthy controls, in line with the limited number of available longitudinal DTI studies [11, 13], progressive white matter degeneration occurred in stroke patients across 3 months.

Interestingly, these lesion-independent early progressive white matter changes were associated with functional recovery, highlighting the incremental value of longitudinal DTI parameters in acute patients to illustrate post-stroke cerebral reorganization and improve prediction of outcome.

In our sample, the most significant association between lesion-independent progressive white matter changes and recovery was observed in the genu of the corpus callosum (CC). We therefore chose to test whether FA of the genu of the CC (baseline scores or progressive changes) would improve prediction of functional outcome above and beyond baseline clinical scores, WMH grade, and infarct volume. Indeed, FA of the genu of the CC independently improved prediction by 23.2% (baseline scores) and 7.6% (progressive change).

We assessed functional recovery by changes in the NIHSS score, which is the gold standard of stroke severity rating and most widely used deficit rating scale in this context [19]. Despite its strong focus on motor function, the NIHSS also includes information on impairment in other domains such as language or attention. This might explain why the CC was found to be the region most significantly associated with recovery. As expected, significant changes were also observed in the CST; however, infarcted tissue frequently overlapped with the CST, and our lesion-independent analytical approach therefore likely will have favored identification of remote changes in white matter integrity.

Previous studies also highlighted the importance of remote changes in callosal white matter integrity post-stroke [12, 14, 20–23]. Gupta et al. (2006) investigated eight patients with large middle cerebral artery strokes at three time points (6, 8, and 12 weeks) and reported callosal decreases in FA suggestive of Wallerian degeneration. Radlinska et al. (2012) showed that stroke-induced lesions do not only lead to a degeneration of the CST fibers within the lesioned hemisphere, but also result in destructive processes of transcallosal fibers, which might in turn ultimately influence cortical reorganization and recovery. In line with this notion, Wang et al. observed FA reductions in the ipsilesional CST and CC to correlate with impaired motor function and increased bilateral fMRI activation during hand movement [24]. Such remote changes are likely related to network affection and might be a promising target for rehabilitation.

Disrupted interhemispheric communication represents a central feature of stroke [25]. We observed reductions in white matter integrity of the body of the CC connecting the primary motor areas; however, significant associations with functional recovery were primarily present in the genu of the CC. Interestingly, Mang et al. (2015) also found that prefrontal transcallosal FA values were significantly associated with motor impairment in chronic stroke patients, even when accounting for age, post-stroke duration, lesion volume, and lesioned CST FA [26]. They concluded that prefrontal regions play an important role in compensating post-stroke motor function by increasing attentional demand, supporting motor learning, and filtering incoming sensory information.

The diagnosis of an acute ischemic infarction, where the damage of brain tissue may still be reversible, enables selection of appropriate treatment and contributes to a more favorable outcome [27]. Recent studies demonstrated a high efficacy of reperfusion therapies (intravenous thrombolysis and/or thrombectomy) in acute ischemic stroke patients regarding improvement in recovery [28–30]. Therefore, we focused on stroke patients with middle cerebral artery infarction receiving this gold standard treatment. In contrast to another study [31] reporting 24 h post-stroke and thrombolysis, axial diffusivity is the most appropriate diffusion metric to quantify damage and predict outcome; in our study, FA was the most sensitive marker.

FA is highly sensitive to microstructural white matter changes, but not very specific to the type of change [6, 32]. FA reflects the degree of diffusion anisotropy within a voxel determined by fiber diameter and density; it is associated with the degree of myelination, extracellular diffusion, inter-axonal spacing, and intravoxel fiber tract coherence [33]. Reduced FA and high MD do not allow us to discriminate between axonal and myelin damage. However, AD is the primary correlate of axonal damage and fiber coherence, whereas RD appears to be modulated by myelin in white matter and fiber integrity [10, 31].

Our results therefore suggest that the observed decreases in FA (along with increases in MD and RD) are indicative of lower myelination and fiber density [32, 34].

It is noteworthy that besides post-stroke decreases in white matter integrity, also pre-existing chronic injury of white matter, as usually assessed by white matter hyperintensity burden, might affect poor post-stroke outcome [35]. However, in our sample, only 10 patients had moderate to severe WMH grades (severe WMH: *N* = 4) WMH grades, and WMH grade was neither associated with stroke outcome, nor did an exclusion of the 4 patients with severe WMH affect results of our regression analyses.

Given a recent suggestion of the American Statistical Association, which emphasizes that scientific conclusions should not be based only on whether a *p* value passes a specific threshold, we also report trends and significant results before Bonferroni-correction in our results section [36].

Some limitations regarding our study are important to consider. First, we decided to use changes in the NIHSS score to assess functional recovery. Although NIHSS has a strong focus on motor function, as also underlined by strong correlations between the mRS and NIHSS (*r* = 0.752, *p* < 0.0001) in our sample, our outcome measure is not exclusively focusing on motor function, making comparisons with studies with specific motor assessments difficult. Those studies were commonly performed at later stages of stroke, thus not including acute patients. However, especially in the acute phase of stroke, a more refined assessment of motor function, such as the frequently used Action Research Arm Test or Wolf Motor Function Test, is frequently not feasible. Secondly, as we only focused on patients with reperfusion treatment to increase homogeneity of our sample, generalization of our results to the whole spectrum of ischemic stroke patients is limited. Thirdly, given comparability of baseline NIHSS and mRS scores and stroke recovery, we combined left and right hemispheric strokes for our analyses. However, it has to be noted that the side of the lesioned hemisphere could affect stroke outcome in the acute and convalescence phase. Fourth, our patients did not receive standardized study-specific rehabilitation which should be considered when interpreting our results. However, all had received rehabilitation by a team of experts while they were inpatients in our university clinic. In addition, the majority (*N* = 20, 66.7%) underwent standard rehabilitation after discharge from our hospital (4–6 weeks at a dedicated rehabilitation center). Seven patients (23%) reported to take part in an outpatient rehabilitation (10 sessions in 4–6 weeks) and three patients did not participate in any dedicated rehabilitation program. Furthermore, it should be noted that imaging studies only can report structural abnormalities and do not necessarily provide an accurate delineation of physiological derangement that occurs after stroke.

## Conclusion

The present longitudinal DTI study in acute stroke patients receiving reperfusion therapy highlights the importance of early lesion-independent progressive white matter changes in relation to functional recovery. DTI represents a useful biomarker to explore widespread post-stroke reorganization in the brain.

## Electronic Supplementary Material

ESM 1 (PDF 39 kb)
