# Supplementary material for: Early Progressive Changes in White Matter Integrity Are Associated with Stroke Recovery
Source: Transl Stroke Res. 2020 Mar 4;11(6):1264–72. doi: 10.1007/s12975-020-00797-x (PMC7575507; doi:10.1007/s12975-020-00797-x)
Supplement: Supplementary file 1 — (PDF 39 kb) [file 12975_2020_797_MOESM1_ESM.pdf]

Early progressive changes in white matter integrity are associated with stroke recovery

Translational Stroke Research

Daniela Pinter (PhD)[1,2]; Thomas Gattringer (MD, PhD)[2,3]; Simon Fandler-Höfler (MD)[2]; Markus Kneihsl (MD)[2]; Sebastian Eppinger (MD)[2]; Hannes Deutschmann (MD)[3]; Alexander Pichler (MD)[2]; Birgit Poltrum (MD)[2]; Gernot Reishofer (PhD)[3]; Stefan Ropele (PhD)[2]; Reinhold Schmidt (MD)[2]; and Christian Enzinger (MD)[1,2,3]

1 Department of Neurology, Research Unit for Neuronal Plasticity and Repair, Medical University of Graz, Graz, Austria

2 Department of Neurology, Medical University of Graz, Graz, Austria

3 Department of Radiology, Division of Neuroradiology, Vascular and Interventional Radiology, Medical University of Graz, Graz, Austria

Corresponding author: Daniela Pinter, PhD, Department of Neurology, Research Unit for Neuronal Plasticity and Repair, Medical University of Graz, Auenbruggerplatz 22, 8036 Graz, Austria, Phone: 0043 316 385 31215, Email: daniela.pinter@medunigraz.at; ORCID: 0000-0003-3138-5225

Table S1. Baseline and Follow-Up comparison of FA, MD, AD and RD values (Median, IQR) extracted from region of interest in patients vs controls. CC = corpus callosum, LCST = left corticospinal tract, RCST = right corticospinal tract. Mann-Whitney U Test. Bonferroni-adjusted level of significance = 0.01.

**Baseline FA values**	**Patients**	**Controls**	**p-value**
CC genu	0.697 (0.058)	0.700 (0.041)	0.279
CC body	0.690 (0.052)	0.707 (0.040)	0.077
CC splenium	0.786 (0.038)	0.794 (0.019)	0.293
LCST	0.594 (0.037)	0.593 (0.027)	0.380
RCST	0.594 (0.034)	0.600 (0.031)	0.885
**Baseline MD values**	**Patients**	**Controls**	**p-value**
CC genu	0.00078 (0.00008)	0.00077 (0.00005)	0.173
CC body	0.00081 (0.00007)	0.00079 (0.00005)	0.030
CC splenium	0.00070 (0.00005)	0.00069 (0.00002)	0.150
LCST	0.00071 (0.00004)	0.00070 (0.00002)	0.992
RCST	0.00070 (0.00005)	0.00070 (0.00002)	0.961
**Baseline AD values**	**Patients**	**Controls**	**p-value**
CC genu	0.00155 (0.00011)	0.00154 (0.00007)	0.267
CC body	0.00162 (0.00009)	0.00159 (0.00005)	0.206
CC splenium	0.00156 (0.00007)	0.00154 (0.00007)	0.267
LCST	0.00123 (0.00007)	0.00124 (0.00006)	0.302
RCST	0.00124 (0.00009)	0.00124 (0.00006)	0.900
**Baseline RD values**	**Patients**	**Controls**	**p-value**
CC genu	0.00041 (0.00009)	0.00039 (0.00004)	0.186
CC body	0.00042 (0.00007)	0.00038 (0.00004)	0.026
CC splenium	0.00029 (0.00005)	0.00028 (0.00002)	0.206
LCST	0.00044 (0.00004)	0.00043 (0.00003)	0.678
RCST	0.00043 (0.00004)	0.00042 (0.00003)	0.595
**Follow-Up FA values**	**Patients**	**Controls**	**p-value**
CC genu	0.671 (0.056)	0.707 (0.034)	0.028
CC body	0.682 (0.039)	0.721 (0.028)	**0.001**
CC splenium	0.789 (0.027)	0.791 (0.021)	0.738

LCST	0.576 (0.036)	0.596 (0.018)	0.055
RCST	0.592 (0.025)	0.599 (0.019)	0.361
**Follow-Up MD values**	**Patients**	**Controls**	**p-value**
CC genu	0.00084 (0.00017)	0.00079 (0.00006)	**0.001**
CC body	0.00086 (0.00020)	0.00078 (0.00005)	**0.001**
CC splenium	0.00076 (0.00026)	0.00069 (0.00003)	**0.003**
LCST	0.00074 (0.00078)	0.00071 (0.00002)	**0.002**
RCST	0.00075 (0.00018)	0.00070 (0.00002)	0.012
**Follow-Up AD values**	**Patients**	**Controls**	**p-value**
CC genu	0.00158 (0.00008)	0.00155 (0.00008)	0.067
CC body	0.00163 (0.00011)	0.00160 (0.00005)	0.060
CC splenium	0.00158 (0.00012)	0.00155 (0.00006)	0.064
LCST	0.00124 (0.00012)	0.00125 (0.00005)	0.485
RCST	0.00126 (0.00008)	0.00123 (0.00005)	0.112
**Follow-Up RD values**	**Patients**	**Controls**	**p-value**
CC genu	0.00048 (0.00023)	0.00037 (0.00004)	**<0.0001**
CC body	0.00047 (0.00029)	0.00038 (0.00005)	**0.001**
CC splenium	0.00035 (0.00032)	0.00028 (0.00002)	**0.002**
LCST	0.00051 (0.00013)	0.00044 (0.00003)	**0.001**
RCST	0.00050 (0.00025)	0.00042 (0.00003)	**0.008**

Table S2: Correlations of cross-sectional extracted white matter integrity and stroke recovery (delta NIHSS). LCST = left corticospinal tract, RCST = right corticospinal tract; CC = corpus callosum. Level of significance after Bonferroni correction = 0.01.

**Baseline FA**	**Stroke recovery**	**p-value**
LCST	-0.135	0.426
RCST	0.048	0.776
CC genu	0.404	**0.013**
CC body	0.204	0.226
CC splenium	0.194	0.250
**Follow-Up FA**		
LCST	-0.164	0.386
RCST	-0.136	0.473
CC genu	-0.117	0.538
CC body	-0.012	0.950
CC splenium	0.176	0.352
**FA change**		
LCST	-0.085	0.654
RCST	-0.186	0.326
CC genu	-0.390	**0.033**
CC body	-0.241	0.199
CC splenium	-0.019	0.922
**Baseline MD**	**Stroke recovery**	**p-value**
LCST	-0.056	0.744
RCST	-0.104	0.540
CC genu	-0.337	**0.041**
CC body	-0,167	0.323
CC splenium	-0.199	0.237
**Follow-Up MD**		
LCST	-0.073	0.701
RCST	0.121	0.523
CC genu	0.002	0.993
CC body	0.136	0.474
CC splenium	0.030	0.874

**MD change**		
LCST	0.035	0.856
RCST	0.125	0.511
CC genu	0.214	0.256
CC body	0.206	0.275
CC splenium	0.127	0.502
**Baseline AD**	**Stroke recovery**	**p-value**
LCST	-0.059	0.728
RCST	-0.174	0.304
CC genu	-0.065	0.702
CC body	-0.062	0.716
CC splenium	-0.224	0.183
**Follow-Up AD**		
LCST	-0.235	0.212
RCST	0.036	0.868
CC genu	0.089	0.639
CC body	0.053	0.782
CC splenium	0.029	0.877
**AD change**		
LCST	-0.304	0.102
RCST	0.184	0.331
CC genu	-0.001	0.994
CC body	-0.017	0.929
CC splenium	0.104	0.584
**Baseline RD**	**Stroke recovery**	**p-value**
LCST	0.079	0.641
RCST	-0.121	0.474
CC genu	-0.381	**0.020**
CC body	-0.218	0.195
CC splenium	-0.208	0.216
**Follow-Up RD**		
LCST	0.124	0.513
RCST	0.110	0.562

CC genu	0.007	0.969
CC body	0.122	0.522
CC splenium	0.064	0.738
**RD change**		
LCST	0.108	0.571
RCST	0.137	0.471
CC genu	0.208	0.271
CC body	0.244	0.193
CC splenium	0.106	0.579
